# The impact of off‐centered positioning on cone beam CT image quality for image‐guided brain SRS treatment

**DOI:** 10.1002/acm2.70388

**Published:** 2025-11-25

**Authors:** Noor Mail, Fang Li, Ron Lalonde, M. Saiful Huq

**Affiliations:** ^1^ Department of Radiation Oncology University of Pittsburgh School of Medicine and UPMC Hillman Cancer Center Pittsburgh Pennsylvania USA

**Keywords:** Cranial metastases, CBCT, Image‐quality and patient alignment‐accuracy, Off‐centered‐Iso‐center, scatter‐to‐primary‐ratio, SRS

## Abstract

**Purpose/objectives:**

Cone beam computed tomography (CBCT) is widely used in image‐guided positioning for patients with brain metastases during stereotactic radiosurgery (SRS). A major issue with CBCT is the decrease in image quality due to scatter, especially when the isocenter is off‐centered for treating peripheral‐metastases. This affects image clarity and CT numbers in skull‐reconstruction. The study aims to measure the scatter‐to‐primary ratio in projection‐images based on gantry‐angle for off‐centered isocenter. It also examines the effects of scatter on CBCT image quality and alignment‐accuracy for off‐centered isocenter.

**Materials/methods:**

A Catphan‐600 phantom was imaged using the head protocol across five different FOV(2–18 cm in SI dimension) to analyze the effect of scatter on CBCT image quality in both centered and off‐centered isocenter Positions. Scatter‐fractions were measured for the Catphan‐600 relative to Field of View (FOV) size and for a head‐phantom in relation to gantry rotation at an off‐centered isocenter. Computed tomography (CT) and CBCT images alignment of the head phantom was compared between centered and off‐centered isocenter placements.

**Results:**

Increasing FOV from 2 to 18 cm significantly reduced image quality; the contrast‐to‐noise ratio (CNR) decreased by 1.7 times with this FOV increase. CNR decreased by 3 times at a 6 cm off‐center position and the skull CT‐number decreased by 350 HU. For the Catphan‐600 at an 18 cm FOV, the scatter‐to‐primary ratio reached 0.67. The scatter‐fraction for the head phantom was 1.8 times higher when fully in‐field compared to partially in‐field. The mean alignment accuracy for the head phantom was 0.3 ± 0.2 mm for isocenter and 0.8 ± 0.3 mm for off‐center isocenter.

**Conclusion:**

Quantitative analysis of CBCT image quality in phantoms reveals substantial image degradation with larger FOV and off‐centered isocenter positioning. The methods in this study provide insights and a framework for refining scatter correction algorithms to achieve precise scatter adjustments. These developments can be used to enhance CBCT image quality and improve target‐alignment‐accuracy.

## 1 INTRODUCTION

Patients with brain metastases are frequently positioned using cone‐beam computed tomography (CBCT) on TrueBeam Linacs for stereotactic radiosurgery (SRS)^1^, ^2^, ^3^, ^4^, ^5^ treatment. However, maintaining image quality (IQ) of CBCT in general is challenging due to image degradation from X‐ray scatter.^6^, ^7^, ^8^, ^9^ This issue is particularly pronounced for off‐centered isocenter positioning, where the patient's head partially moves in and out of the beam during gantry rotation, leading to a significant reduction in image quality, especially in the accuracy of skull's computed tomography (CT) numbers. This is because existing image reconstruction methods, such as filtered back projection, rely on the assumption that scattered photons are not detected; the existence of scatter violates these assumptions and results in inaccurate scatter correction. The quantification of scatter signal and its variation as a function of gantry rotation for Off‐centered isocenter position depends on the Mets location in brain.

Large cone angles increase the impact of scatter, which in turn impacts image contrast and CT number accuracy.^10^, ^11^, ^12^, ^13^, ^14^, ^15^, ^16^, ^17^ Conventional scatter control techniques, including increased air gap,^18^ bowtie filters,^6^ antiscatter grids,^19^ and hybrid methods,^20^ have been explored to mitigate scatter artifacts. Advances in scatter correction, such as empirical technique,^20^, ^21^ analytical technique,^22^, ^23^ and Monte Carlo^24^, ^25^, ^26^ offer promising improvements but still face challenges in achieving optimal results. Scatter signal measurements across different field of view (FOV) and off‐center isocenter positions for brain have not been fully reported, nor has their impact on CBCT image quality been thoroughly explored.

To fill this gap, detailed analysis and definitions of CBCT artifacts are required to enhance our understanding of imaging performance and identify effective solutions. Further studies are necessary to develop innovative scatter rejection devices and accurate scatter correction models based on precise scatter measurements, particularly for off‐centered isocenter CBCT scans of brain metastases which demand alignment accuracy within 1 mm.

In this study, the degree of CBCT image quality degradation in a clinical CBCT system on True‐Beam 2.7 (Varian Medical Systems, Palo Alto, CA) is assessed for the first time as a function of FOV and off‐centered isocenter (e.g., brain metastases near the skull perimeter). We quantify scatter signal measurements in projection images for both centered and off‐centered isocenter positions. Specifically, we aim to quantify the scatter signal across projections, assess scatter variation for off‐centered brain isocenter, and evaluate the impact of scatter variation on CBCT image quality using metrics such as contrast‐to‐noise ratio (CNR) and CT number accuracy in skull reconstruction. Additionally, we quantify the impact of off‐centered isocenter location on CBCT alignment accuracy.

## 2 METHOD AND MATERIAL

### 2.1 Scatter to primary ratio: Cerrobend cylinder

**FIGURE 1:**
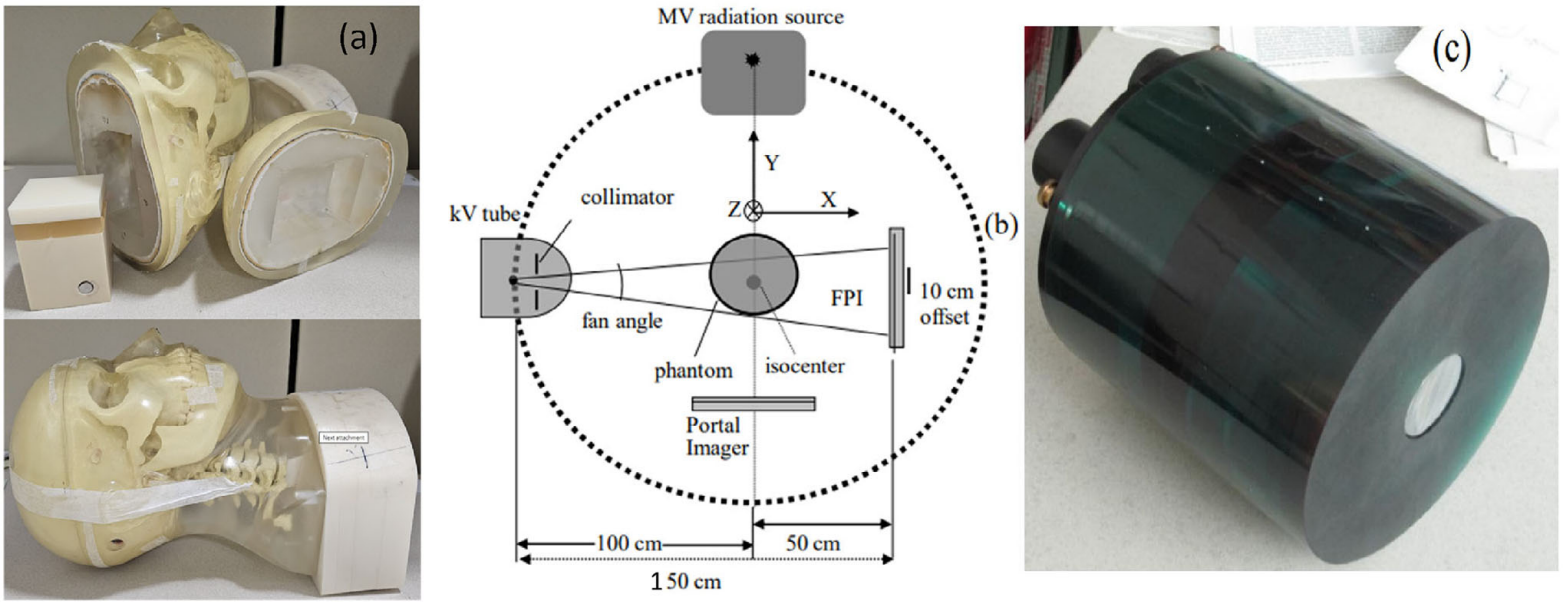
(a) Picture of the Lucy anthropomorphic Head Phantom, with a Lucite cube centered in its brain, 12 mm diameter and depth of lateral hole near corner to insert a Cerrobend cylinder (b) A schematic of the CBCT geometry for the phantom placed laterally off‐center and (c) Catphan‐600 phantom. The beam‐blocker's objectives were to block the primary beam and measure the scatter beam in the projection images.

The Lucy Phantom, an anthropomorphic CyberKnife head phantom from Standard Imaging (Wisconsin, USA) was used in this study shown in Figure 1a. It features a detachable Lucite cube centered in its brain, a 12 mm diameter and 12 mm depth lateral hole was drilled at one corner to insert a Cerrobend cylinder (CC) as a beam‐blocker to measure the scatter beam in the projection images. Regardless of gantry angle, the CC was set to appear square in the projection images. A GE LightSpeed 16Slice CT simulator with a slice thickness of 1.25 mm, tube voltage of 120kVp, and exposure of 300mAs was used to simulate the phantom. Positioning marker was drawn with the help of external laser of the CT simulator to ensure precise head phantom prepositioning. The purpose of these crossline markers in the lateral and forehead was to reposition the phantom at the treatment Isocenter for CBCT scans. All CBCT scans were acquired using the standard head full fan bowtie filter (100 kVp, 150 mAs, 25 cm^2^ field of view, gantry rotation range of 200‐220°). The amorphous‐silicon flat panel X‐ray imaging detector (Varian‐Medical‐System, Palo Alto, California) was utilized. It has a 0.055‐mm‐thick CsI:Tl X‐ray converter and a 2048×1536 array of 0.2× 0.2 mm2 pixels. The largest area of the detector's image is 40×30 cm^2^. For every pixel, an a‐Si: H photo diode with an 82% geometric fill factor is connected to an a‐Si: H thin‐film transistor. The system geometry gives a source‐to‐isocenter distance of 100 cm and source‐to‐detector distance 150 cm. For the CBCT scans, the clinical protocol for brain SRS was used. This involves rotating the gantry from 20° to 220° and obtaining 500 projection images during the gantry rotation of 200°. Scans were taken with three different CC beam‐blocks (5 mm, 8 mm, and 12 mm diameters, each with corresponding lengths) inserted sequentially into the phantom. The beam‐blocker's objectives were to block the primary beam and to quantify the scatter signal in the projection images. Due to the off‐centering isocenter positioning at the CC beam‐block, the phantom was fully in the active field for certain range of the gantry angle and partially out at specific gantry angle from the active X‐ray field. Changes in the phantom volume in the active X‐ray field as a function of gantry angle result in variations in the scatter signal as a function of projection images acquired during the CBCT scan. Reconstruction was performed using Filtered back‐projection (FBP), which employs the Feldkamp‐Davis‐Kress (FDK) approach after adding a kernel‐based scatter correction to the 2D projection images, that is used by the Varian TrueBeam 2.7 CBCT system. It does not have a more advanced approach to model the scatter behavior in a 3D volumetric image.

Cone beam computed tomography (CBCT) images were automatically registered to planning CT images using Varian's TrueBeam system, which uses a mutual information (MI)‐based algorithm to provide CT and CBCT alignment. The CC beam‐block's center was designated as the isocenter, keeping its image position stable across all 500 gantry‐angle projections, thereby facilitating the extraction of primary around the CC and scatter signals within the CC image shade using a MATLAB script.

#### 2.1.1 Scatter to primary ratio: head phantom

The scatter‐to‐primary ratio (SPR) was calculated for each of the 500 projection images acquired at various gantry angles for the off‐centered isocenter of the head phantom's CBCT scan. The beam block technique methodology was used to determine the SPR, which is the ratio of scattered to primary radiation that reaches the imager. This method blocked the primary beam using a Cerrobend beam‐blocking material while allowing scattered radiation to reach the detector. The shade of the beam blocker in the projection image was quantified as scattered beam signal and the primary signal was quantified from near the neighbor area around the beam block's shade. The dark and gain corrections have been applied before acquiring the CBCT scans. The dark signal was carefully checked in the imager center in acquiring 50 dark field images, it was negligible and subtracted from the signal behind the block (scatter) and signal near the block (primary + scatter). All of the CBCT scans' projection images were saved locally on a laptop for Matlab analysis after being exported as Dicom images from Varian True Beam service mode. Three CC with different diameters and lengths were fabricated, inserted one at a time for the CBCT scan, and placed at the phantom's off‐center isocenter in order to determine the scatter fraction. These CC beam‐blocks appeared square in projection images, and their stable position made it straightforward to quantify the scatter signal in MATLAB across projections. The SPR was defined as:

(1) SPR=S/P

where S represents the average scatter signal (5 x 5 pixels) behind the Cerrobend beam‐block on the flat‐panel imager and P represents the primary radiation signal, which was obtained by subtracting the scatter from the primary + scatter signal near the beam block. It should be noted that the transmission for the 5 mm thick slab was checked separately for 120 kVp, which was incredibly low. Based on this, it is believed that the Cerrobend beam‐block did not have a transmission, mean no primary beam signal behind the beam‐block. Three CBCT scans were taken for each cylinder and SPR was then plotted against CC beam‐block size for each projection. Scatter fraction (SF) was determined through linear fitting:

(2) SF=m×X+C

where m is the slope, X is the Cerrobend beam‐block size, and C is constant. This allowed SF quantification across 500 projections to assess scatter variation by gantry angle.

#### 2.1.2 Scatter to primary ration: Catphan‐600

A beam‐blocker method^9^ was used to evaluate the SPR for five FOV (ranging from 2 to 18 cm) under the same imaging conditions. For SPR measurement, a 5×5 mm^2^ Cerrobend beam‐block (4 mm thick) was placed on the central axis before the phantom on the side facing the source. 15 images of the Catphan phantom were acquired for five different FOV with a bowtie filter at 100 kVp and 150 mAs. The scatter signal was measured behind the Cerrobend beam‐block (5×5 pixels in the projection image), while the primary radiation signal was taken near the beam‐block shadow. The SPR was calculated across five FOV (2‐18 cm) in the superior‐inferior direction.

### 2.2 Cone‐beam CT image quality

The Catphan‐600 phantom (The Phantom Laboratory, Salem, NY) shown in Figure 1c was used to assess X‐ray scatter's effect on CBCT image quality. Images were acquired using the head standard protocol across five FOV (2‐18 cm, SI), at 100 kVp and 150 mAs, examining scatter effects for centered and off‐centered isocenter positions. All scans were reconstructed with a 1.25 mm slice thickness. To evaluate the impact of scatter and off‐centered isocenter on CBCT quality, images were analyzed under both low‐ and high‐scatter conditions. The Feldkamp‐Davis‐Kress (FDK) algorithm^27^ was used for 3D backprojection in CBCT reconstruction. CT images were acquired on a GE Light Speed 16‐slice CT simulator (GE, Waukesha, Wisconsin, USA) with a 1.25 mm slice thickness. Image quality parameters such as CNR and CT number accuracy were used to assess skull bone reconstruction. The FOV was lowered from 18 to 2 cm in order to imitate low scatter circumstances and reduce scattering effects. The collimator size was reduced along with the superior‐inferior directions for a desired FOV, whic is the scanning length or beam projection length at the isocenter.

#### 2.2.1 Contrast‐to‐Noise Ratio (m_CNR_)

The contrast‐to‐noise ratio (mCNR) was analyzed from volumetric images of the Catphan phantom for the acrylic inserts at several FOV. Acrylic was chosen for the CNR measurement because its density is similar to that of soft tissue and water. A 100 mm^2^ circular region of interest was selected on the acrylic insert and the surrounding background in order to quantify contrast and noise. The standard deviation of the noise in the surrounding background region (∼100 mm^2^) and the noise in the acrylic (∼100 mm^2^) were taken into account. The CNR was calculated as:

(3) $$m_{CNR}=\frac{\left[\bar{\mathrm{CT}\#}\left(H_{2}O\right)-\bar{\mathrm{CT}\#}\left(\mathrm{Acrylic}\right)\right]}{\left(\frac{\bar{\mathrm{Noise}}\left(H_{2}O\right)+\bar{\mathrm{Noise}}(\mathrm{Acrylic}}{2}\right)}$$

where the mean voxel values in water and acrylic are denoted by CT#(H_2_O) and CT#(Acrylic), respectively and the standard deviation of the voxel values in water and acrylic is denoted by Noise(H_2_O) and Noise(Acrylic).

#### 2.2.2 CT number accuracy: skull and teflon

We used a head phantom and a catphan phantom to test the impact of FOV and Isocenter location on CT number accuracy. A square ROI (∼49 pixels2) of an axial image slice was measured using Matlab (2020) to determine the average CT numbers within the image (inserts). The CT number for the skull obtained using a CT simulator with a line profile across the skull was compared to the CT number determined from the CBCT images of the head phantom.

### 2.3 CBCT alignment accuracy

The head phantom was prepositioned with the help of an infrared optical system on a 6D couch using a QFix mask and support. CBCT scan was acquired at the isocenter position using a standard head full fan protocol (100 kVp, 150 mAs, 25 cm^2^ FOV) and reconstructed with a 1 mm slice thickness. A mutual information (MI)‐based algorithm was used to register CBCT to the planning CT, which includes the full skull, enabling online auto‐matching. After the 6D alignment, the couch shift was applied and recorded. For the off‐center positioning accuracy test, a known right lateral shift of 2, 5, and 8 cm was applied to simulate clinical scenarios where peripheral targets require the head to be off‐centered. CBCT scans were taken at off‐center isocenter locations, and the alignment discrepancies following CBCT and CT fusion, known as residual 6D couch shifts, were recorded. The residual 3D couch shift vector sum was calculated for all three off‐center isocenter coordinates.

## 3 RESULTS AND DISCUSSION

### 3.1 Scatter‐to‐primary ratio measurements

#### 3.1.1 Head phantom

**FIGURE 2:**
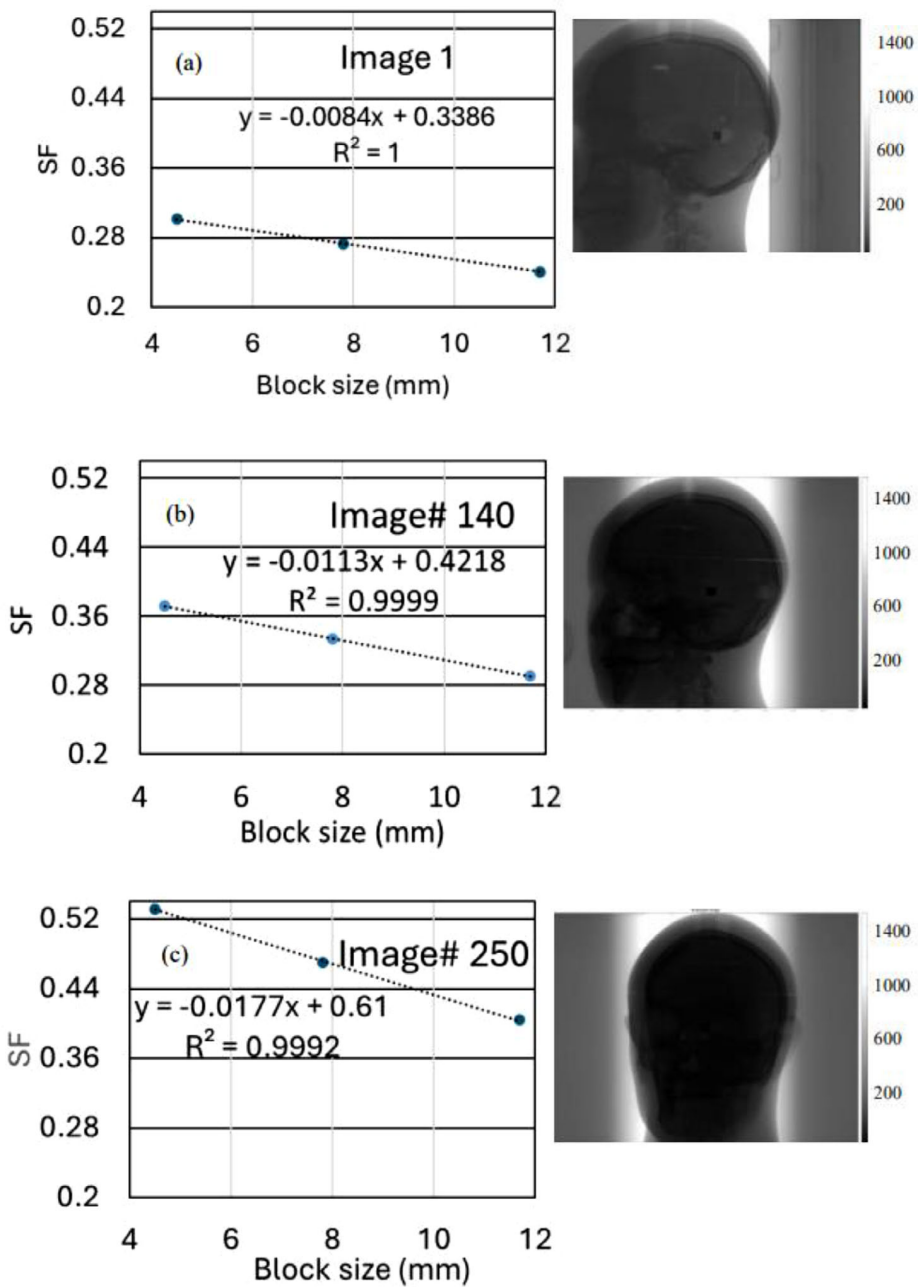
Scatter‐to‐primary ratios (SPR) shown against Cerrobend cylinder (CC) beam‐block size, projection images are shown on the right. (a) Image#1: the phantom is somewhat outside the X‐ray field, (b) Image#140: phantom is partially inside the field, and (c) Image #250, the phantom is completely inside the field.

**FIGURE 3:**
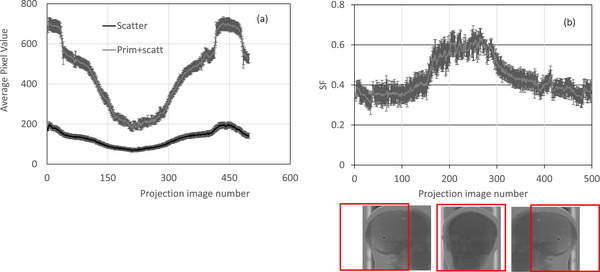
(a) shows scatter and primary signals as a function of projection images, captured across various gantry angles with an 8 mm beam‐block. (b), Scatter‐fraction is plotted across 500 projections.

Figures 2a–c illustrate the SPR for three projection images taken at different gantry angles. In Figure 2c, a higher SPR was observed, indicating the entire head phantom was within the X‐ray field. Figure 2c shows a higher slope than Figures 2a and b, resulting in a larger extrapolated scatter fraction (SF) value of 0.61 compared between 0.338 and 0.42 for Figures 2a and b, respectively. This lower SF in Figure 2a reflects the head phantom's is partially exposed in the X‐ray field. Given the off‐center isocenter setup, the phantom is sometimes partially within and outside of the X‐ray field at different gantry angles. Figure 3a shows scatter and primary signals behind and near the CC beam‐block as a function of projection number, captured across various gantry angles with an 8 mm beam‐block. Due to the phantom's anthropomorphic structure, the primary signal clearly displays the variation due to bony and heterogeneous areas, while scatter behind the CC beam‐block varies similarly with changes in gantry angle. Projection numbers 200‐350 exhibit a high scatter relative to the primary beam when the head phantom is fully in the X‐ray field. In Figure 3b, SF is plotted across 500 projections, revealing scatter fraction variation with projection number. At angles where the head phantom is partially out of the X‐ray field (projections 1‐150 and 450‐500), lower SF values were recorded. At other angles, the phantom is fully in the X‐ray field, resulting in a higher SF as seen in projections 200‐400.

#### 3.1.2 Catphan Phantom

**FIGURE 4:**
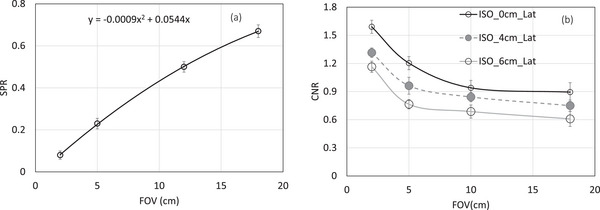
Illustrates scatter‐to‐primary (SPR) measurements for Catphan phantom as a function of the field of view (FOV) for Isocenter positioned placed centrally.

For the Catphan phantom, Figure 4 illustrates SPR measurements as a function of the FOV for isocenter position placed centrally. SPR increased with FOV size, rising from 0.10 at a 2 cm FOV to 0.67 ± 0.0544 at an 18 cm FOV, following a polynomial fit (SPR = aX^2^ + bX, with coefficients between −0.0009 and 0.0544, respectively). This is a nonlinear behavior, which explains how the SPR rises with FOV size, is captured by the polynomial fit. According to our findings, scatter contributes significantly to the overall detector signal at the largest FOV.

### 3.2 CBCT Image Quality

#### 3.2.1 Contrast‐to‐Noise Ratio (m_CNR_)

**FIGURE 5:**
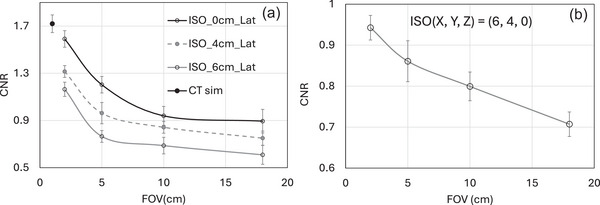
(a) displays the contrast‐to‐noise ratio (CNR) data for an acrylic insert in Catphan phantom as a function of FOV and Isocenter position. For comparison, the CNR from CT Sim is also displayed as a single data point. (b) Displays the Catphan phantom's analyzed CNR (Acrylic insert) as a function of FOV at an off‐center ISO position (6,4,0).

Figure 5a displays the CNR data for an acrylic insert as a function of FOV, as determined from the scan of a Catphan phantom. Image quality is decreased when the FOV is increased from 2 to 18 cm; more precisely, the CNR decreased by a factor of 1.77 when the FOV was increased from 2 to 18 cm at isocenter. For the off‐center isocenter between 4 and 6 cm in the lateral direction, the CNR was reduced by a factor between 1.88 and 1.94, respectively. This is due to the filtered back‐projection (FBP) feature of the Varian TrueBeam 2.7 CBCT system, which applies a kernel‐based scatter correction to the 2D projection images before using the Feldkamp‐Davis‐Kress (FDK) approach. It does not have a more sophisticated approach to model the scatter behavior in a 3D volumetric image.

Since with larger FOV both the contrast and voxel noise are reduced due to higher scatter signal, the effect of X‐ray scatter on the m_CNR_ is a ratio of two reducing quantities: degradation in contrast and noise. The reduction in contrast with scatter is higher than reduction in voxel noise; therefore, the net effect is the degradation in m_CNR_ as quantitatively shown in Figure 5a. Increasing the FOV up to 18 cm significantly reduces the CNR. The shading artifact throughout the image especially between the center and contrast inserts reduces with the decrease in FOV resulting in an improvement in low contrast visibility. The analyzed m_CNR_ (acrylic insert) of the Catphan phantom is shown in Figure 5b as a function of FOV for an off‐center isocenter position (0,4,6). CNR drops by a factor of 1.5 with increasing FOV (from 2 to 18 cm).

#### 3.2.2 CT Number Accuracy

**FIGURE 6:**
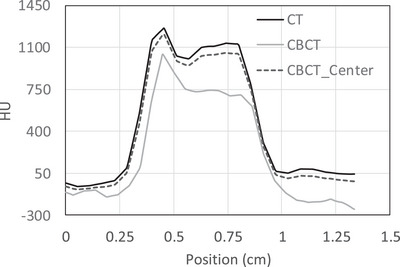
The skull profiles for the Cone beam computed tomography (CBCT) off‐center ISO and CT sim images reveal reduced CT values and a 0.5 mm decrease in left‐hand skull size.

CT and CBCT alignment for treating cranial Mets is based on skull‐to‐skull alignment. Therefore, skull reconstruction accuracy in CBCT images is crucial, including skull CT number and profile comparison. For comparison, the Skull and Teflon CT number are listed in Table 1. Skull CT numbers in CBCT for head phantoms were 15 HU and 330 HU lower, respectively, than CT simulation results at the center and off‐center isocenter. The skull profiles for the CBCT‐center, CBCT off‐center Isocenter and CT sim images are shown in Figure 6. The CBCT off‐center profile shows lower CT values and a 0.5 mm reduction in skull size on the left. Teflon CT numbers for Catphan phantom scans were similarly lower between −5 HU and 225 HU for centered and off‐center isocenter compared to CT sim values.

**TABLE 1:** The Skull and Teflon CT numbers are listed for CBCT at different FOV and Isocenter position vs. CT sim.

Technique	Skull_Mean HU	Teflon HU
CT	1700 ±30	975 ±20
CBCT_Center	1685 ±39	980 ±29
CBCT_Off ISO	1369 ±55	750 ±53

Abbreviation: Cone beam computed tomography; CBCT, Field of view; FOV.

#### 3.2.3 CBCT Alignment Accuracy

**FIGURE 7:**
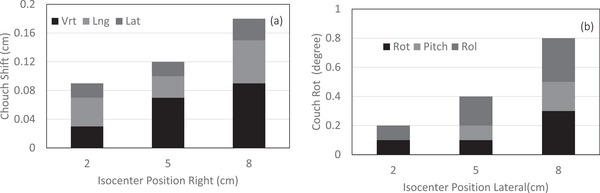
The residual couch shift is plotted against the off‐center ISO location. (a) In all three directions (X,Y,Z) and (b) couch rotation, rolling and pitch.

The 6D couch shift performed in all three directions following the CBCT and CT alignment is displayed against the off‐center isocenter location in Figures 7a and b. As the off‐center isocenter distance increases, so does the couch shift uncertainty. There may be several reasons for the increase in couch shift uncertainty but one of the causes is the decrease in the CT number of the skull bone due to scatter and the possible truncation‐induced decline in skull volume information for the off‐center isocenter position.

**FIGURE 8:**
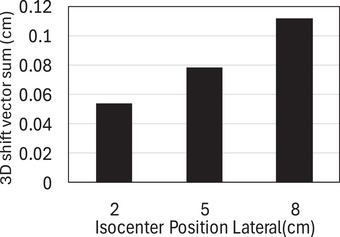
The residual couch shift vector sum (X,Y,Z) against the known off‐center isocenter position.

Figure 8 displays the residual 3D couch shift vector sum (X, Y, Z) vs. the given off‐center isocenter location. As the off‐center Isocenter distance increases, the 3D shift vector total exhibits a linearly rising trend. At an isocenter point 8 cm off‐center, the maximum vector sum was 1.15 mm.

## 4 DISCUSSION AND CONCLUSIONS

This study investigated the SPR for a head phantom at an off‐center isocenter position over 500 projection images per CBCT scan. SPR and scatter fraction (SF) variation was seen in relation to projection images captured at different gantry orientations. The fluctuation in head phantom volume exposed in the X‐ray projection as a function of gantry angle is responsible for the variance in SPR and SF. The results indicate that scatter and its varied nature have a significant influence on the accuracy of skull reconstruction CT numbers. A decrease in skull CT number reported in CBCT images acquired at off‐center isocenter positions of head phantom, revealing that current systems struggle to maintain CT number accuracy which may be attributed to scatter in general and variations in scatter specifically as a function of gantry angle for 500 projection images. These findings highlight the shortcomings of existing reconstruction techniques, which presume ineffective management of scattered photons, suggesting the need for better scatter correction algorithm. It lacks a more sophisticated method for simulating scatter behavior in a 3D volume.

The impact of energy sensitivity on the SPR data in our case might be very minimum because of two reasons, 1) the beam quality reaching the detector is almost pseudo monochromatic as it passes through a bowtie filter and patient, which removes the low energies and The detective quantum efficiency (DQE) for indirect CsI:Ti FPI are not very sensitive to the 120kVp beam energy range employed in CBCT.

It is worth mentioning that scatter for the head phantom and the Catphan phantom might differ because of material differences and size. For the Catphan phantom, two conditions were investigated including low scatter (small FOV) and high scatter (large FOV) at Isocenter and off‐center Isocenter positions. The Catphan Phantom is the only phantom that was uniform and approximately the same size as the head phantom. We attempted to isolate the effects on image quality caused by truncation for the off‐center ISO CBCT and beam hardening for the Isocenter CBCT by comparing between 2 cm FOV and 18 cm FOV and leaving just scatter to observe image quality differences.

In addition, SPR as a function of FOV was obtained for the Catphan phantom at isocenter position placed centrally. SPR as function of FOV exhibits nonlinear behavior, following a polynomial fit. According to our findings, scatter contributes significantly to the overall detector signal at the largest FOV. The influence of scatter on CBCT image quality was assessed by analyzing CNR and CT number accuracy using Catphan phantom at center isocenter position as a function of FOV. Reduction in image quality including CNR and CT number accuracy was seen with the increase in FOV as expected. Furthermore, the dependence of imaging performance on off‐center isocenter position and FOV was explored. Reduction in CNR and CT number accuracy was observed as the off‐center isocenter position is increased. This substantial impact on CT number accuracy and CNR may be attributed to the lack of sophisticated approach of the filtered back‐projection (FBP) feature which applies a kernel‐based scatter correction to the 2D projection images before using the Feldkamp–Davis–Kress (FDK).

In summary, this study quantified SPR and SF as a function of projection images and evaluated their effects on CBCT image quality, particularly CNR and skull CT number accuracy. Comprehensive assessments of SPR data and the creation of standardized image quality metric for scatter impact can facilitate the assessment of new correction methods and encourage their integration into commercial CBCT systems, potentially enhancing the quality and clinical utility of CBCT imaging. The SPR data as a function of block size exhibited a linear trend throughout all 500 graphs. The linear trend can be attributed to the pseudo monochromatic quality of the beam departing the patient, resulting in uniform lateral scatter. The beam travels through a bowtie filter that is thicker at the edges and thinner in the middle, compensating for the patient's thickness in the center and thinner at the sides, resulting in fluence flatness and the removal of low energy that absorbs in the bowtie filter and patient.

The current Filter‐Back projection reconstruction algorithm^27^ with True Beam version 2.7 was found to be inadequate in terms of scatter correction, especially for CBCT scans obtained at off‐center Isocenter positions. It lacks a more sophisticated method to model the scatter behavior in a 3D volumetric image, suggesting the need for better scatter correction. It will be interesting to test the response of the Elekta Versa and Varian Hypersight CBCT images to scatter correction for the off‐center Isocenter position.

## AUTHOR CONTRIBUTIONS

**Noor Mail**: Project Idea; data collection; data analysis; writing. **Fang Li**: Data collection and analysis. **Ron Lalonde**: Data review; analysis and correction. **M. Saiful Huq**: Data Review and manuscript review/correction.

## CONFLICT OF INTEREST STATEMENT

The authors declare no conflicts of interest.
